# Surgical Management of Stage IIIA Non-Small Cell Lung Cancer

**DOI:** 10.3389/fonc.2017.00249

**Published:** 2017-10-26

**Authors:** Paul E. Van Schil, Lawek Berzenji, Suresh K. Yogeswaran, Jeroen M. Hendriks, Patrick Lauwers

**Affiliations:** ^1^Department of Thoracic and Vascular Surgery, Antwerp University Hospital and Antwerp University, Antwerp, Belgium

**Keywords:** lung cancer, stage III, treatment, chemotherapy, radiotherapy, surgery, multimodality therapy, induction therapy

## Abstract

According to the eighth edition of the tumor–node–metastasis classification, stage III non-small cell lung cancer is subdivided into stages IIIA, IIIB, and IIIC. They represent a heterogeneous group of bronchogenic carcinomas with locoregional involvement by extension of the primary tumor and/or ipsilateral or contralateral lymph node involvement. Surgical indications have not been definitely established but, in general, long-term survival is only obtained in those patients in whom a complete resection is obtained. This mini-review mainly focusses on stage IIIA disease comprising patients with locoregionally advanced lung cancers. Different subcategories of N2 involvement exist, which range from unexpected N2 disease after thorough preoperative staging or “surprise” N2, to bulky N2 involvement, mostly treated by chemoradiation, and finally, the intermediate category of potentially resectable N2 disease treated with a combined modality regimen. After induction therapy for preoperative N2 involvement, best surgical results are obtained with proven mediastinal downstaging when a lobectomy is feasible to obtain a microscopic complete resection. However, no definite, universally accepted guidelines exist. A relatively new entity is salvage surgery applied for recurrent disease after full-dose chemoradiation when no other therapeutic options exist. Equally, only a small subset of patients with T4N0-1 disease qualify for surgical resection after thorough discussion within a multidisciplinary tumor board on the condition that a complete resection is feasible. Targeted therapies and immunotherapy have recently become part of our therapeutic armamentarium, and it might be expected that they will be incorporated in current regimens after careful evaluation in randomized clinical trials.

## Introduction

Precise indications for surgical treatment of stage III non-small cell lung cancer (NSCLC) remain highly controversial although randomized controlled trials have been performed (1). Several reasons account for this ongoing debate. There are several subsets of stage III NSCLC related to the extension of the primary tumor and hilar or mediastinal lymph node involvement (2). Stage III represents an intermediate zone between clearly resectable, early stage disease, and metastatic involvement for which a surgical intervention is only very rarely indicated. There is also a lack of precise definitions that can universally be applied, as, e.g., definition of “resectable” T4 or N2 disease, which is largely dependent on the local expertise (3).

Combined modality therapy is indicated for most patients with stage III NSCLC who have a good performance status but the precise role of surgery, radiotherapy, and chemotherapy within such combined modalities setting has not been firmly established (4). Moreover, with the introduction of targeted agents, and more recently, also immunotherapy, the therapeutic options have clearly expanded. In this mini-review, we mainly focus on stage IIIA with the main emphasis on the contribution of thoracic surgery. Finally, targeted therapies and immunotherapy are mentioned as new therapeutic options that have to be further evaluated, and the relatively new concept of salvage surgery will be highlighted.

## T3N1M0 (Stage IIIA)

T3N1 is relatively rarely encountered in thoracic surgery. Most of these patients are operated for clinical T3 involvement and, incidentally, during the intervention N1 disease is discovered. Every attempt should be made to obtain a complete resection with negative surgical margins (5). In some patients, chest wall resection and reconstruction will be required. Adjuvant chemotherapy is indicated for tumors >5 cm and/or when lymph nodes are involved. In case of microscopic residual disease at the section margins, additional radiotherapy may also be considered after discussion within a multidisciplinary team to decrease the local recurrence rate (6).

## T1-3N2M0 (Stage IIIA–IIIB)

Stage IIIA-N2 remains one of the most controversial areas in thoracic oncology although results of large phase III trials have become available for almost 10 years. N2 involvement represents quite heterogeneous disease entities including unexpected or unforeseen or “surprise” N2 disease, intranodal, and extracapsular invasion, single and multilevel N2 disease, and finally, limited and bulky N2 involvement (7). For this review, we focus on patients with potentially resectable N2 involvement proven by minimally invasive or invasive staging procedures as they represent a highly controversial indication for a surgical intervention. It should already be noted that there is no universally accepted definition of “potentially resectable N2,” which largely depends on the specific center and the experience of the involved thoracic surgeon.

Three large randomized trials have been reported at major meetings and published in highly ranked journals but they don’t provide a definite answer on optimal management of this disease stage (8–10). In the Intergroup (INT) 0139 and the more recent ESPATUE trial, patients were treated with induction chemoradiation and subsequently randomized between surgery or further radiotherapy. In the ESPATUE phase III trial, the induction therapy was quite complicated and consisted of induction chemotherapy followed by chemoradiation. In the European Organization for Research and Treatment of Cancer (EORTC) 08941 trial, only induction chemotherapy was given followed by surgery or radiotherapy in case of response to chemotherapy, also randomizing those patients with a minor response. In all three trials, overall survival was not different between both arms although in the Intergroup trial progression-free survival was better in the group undergoing surgical resection. In the latter study, mortality of pneumonectomy was unacceptably high, especially for those patients undergoing complex, intrapericardial pneumonectomies. An unplanned subanalysis matched patients undergoing lobectomy after induction chemoradiation to a similar group treated by chemoradiation only. A highly significant survival difference was found favoring the surgical arm (9). This made the authors conclude that there is an advantage for surgical intervention on the condition that a complete resection can be obtained by performing a lobectomy after induction therapy. It should also be noted that the EORTC and Intergroup trial were designed at a time when routine positron emission tomographic scanning was not yet incorporated and that staging by minimally invasive techniques was not available in most participating centers.

Several meta-analyses performed on this subject tried to provide more definite answers, but did not reach similar conclusions. A summary with conclusions is provided in Table 1. Two of these meta-analyses should be highlighted. McElnay et al. compared bimodality and trimodality regimens including six trials with a total of 868 patients (11). They concluded that the outcome for the radiotherapy and surgical arms were similar for bimodality regimens, but that there is a 13% survival advantage for surgical intervention within combined trimodality therapy consisting of chemotherapy, radiotherapy, and surgery. This does not reflect a selection bias as in both arms patients qualified for surgical resection. However, the latter difference did not reach statistical significance at the 0.05 level. Conclusions of the most recent meta-analysis including randomized trials that compared surgery with radiotherapy as local treatment modalities were more moderate, stating that there was no difference in overall and progression-free survival between surgery and radiotherapy in the setting of stage III NSCLC (12).

**Table 1:** Recent meta-analyses and systematic reviews on stage III non-small cell lung cancer.

Reference	Number of included studies	Number of randomized studies	Total number of patients	Overall survival	Disease-free survival; progression-free survival	Tumor downstaging; pathological complete response; local control	Toxicity
McElnay et al. (11)	6	6	868	– OS was not significantly different between surgery and radiotherapy in bimodality treatment trials [HR = 1.01 (95% CI 0.82–1.23); *p* = 0.954] – OS was not significantly different between surgery and radiotherapy in trimodality treatment trials [HR = 0.87 (95% CI 0.75–1.01); *p* = 0.068] – Overall OS of all trials [HR = 0.92 (95% CI 0.81 to 1.03); *p* = 0.157]			

Pöttgen et al. (12)	6	6	1,322	– OS was not significantly different between surgical and definitive radiotherapy arms [HR = 0.92 (95% CI 0.82–1.04); *p* = 0.19]	– PFS was not significantly different between surgical and definitive radiotherapy arms [HR = 0.91 (95% CI 0.73–1.13); *p* = 0.4]		– Treatment-related toxicity was higher in the surgical arms than the radiotherapy arms [RR = 3.56 (95% CI 1.65–7.72); *p* = 0.0005]

Xu et al. (13)	7	7	1,049	– OS was not significantly different in the surgical group compared to the radical radiotherapy group after neoadjuvant chemotherapy or chemoradiotherapy [HR = 0.95 (95% CI 0.81–1.10); *p* = 0.49]	– PFS was not significantly different in the surgical group – Compared to the radical radiotherapy group after neoadjuvant chemotherapy or chemoradiotherapy [HR = 0.90 (95% CI 0.77–1.05); *p* = 0.19]	– Mediastinal pCR was significantly different in patients who received neoadjuvant chemoradiotherapy prior to surgical resection compared to those who received neoadjuvant chemotherapy [OR = 3.61 (95% CI 1.07–12.15); *p* = 0.04]	

Guo et al. (14)	12	8	2,724	– 5-year OS was significantly different when comparing induction chemoradiotherapy to induction chemotherapy alone prior to surgery [HR = 0.89 (95% CI 0.68–1.19); *p* = 0.44]	– 5-year PFS was not significantly different when comparing induction chemoradiotherapy to induction chemotherapy alone prior to surgery [HR = 0.74 (95% CI 0.43–1.26); *p* = 0.26]	– Induction chemoradiation prior to surgery results in significantly improved downstaging [OR = 0.75 (95% CI, 0.63–0.89); *p* = 0.001], mediastinal pCR [OR = 0.72 (95% CI, 0.60–0.88); *p* = 0.001], and in LC [OR = 0.64 (95% CI, 0.48–0.85); *p* = 0.002] compared with induction chemotherapy alone	

Ren et al. (15)	3	3	1,084	– 2- and 4-year OS were not significantly different when comparing induction treatment plus surgery [RR = 1.00 (95% CI 0.85–1.17); *p* = 0.98] to combined chemoradiotherapy as definitive therapy [RR = 1.13 (95% CI 0.85–1.51); *p* = 0.39]	– PFS was significantly different when comparing induction chemoradiotherapy prior to surgery [RR = 1.78; (95% CI 1.08–2.92); *p* = 0.02] to chemotherapy alone [RR = 1.05 (95% CI 0.61–1.81); *p* = 0.86]		

Shah et al. (16)	7	1	339	– OS was not significantly different when comparing induction chemoradiotherapy to induction chemotherapy alone after meta-analysis of RCTs [HR = 0.93 (95% CI 0.54–1.62), *p* = 0.81] and retrospective studies [HR = 0.77 (95% CI 0.50–1.19), *p* = 0.24]			

*CI, confidence interval; DFS, disease-free survival; HR, hazard ratio; LC, local control; OR, odds ratio; OS, overall survival; pCR, pathological complete response; PFS, progression-free survival; RCT, randomized controlled trials; RR, relative risk*.

In most studies, it has been clearly demonstrated that downstaging of mediastinal lymph nodes is a major prognostic factor; so, every attempt should be made to thoroughly restage the mediastinum after induction therapy by minimally invasive or invasive techniques before embarking on a major surgical intervention (17–19).

It seems improbable that similar, large-scale phase III trials in patients with N2 disease will still be initiated as currently, many more therapeutic options including targeted therapies and immunotherapy, have become available (20, 21). These newer modalities still have to be evaluated in randomized phase II and phase III trials to determine the optimal combination that provides the best long-term results. As no clear recommendations can be made at the present time, every patient with N2 disease has to be carefully evaluated by a multidisciplinary thoracic oncological team including experienced thoracic surgeons to determine the optimal diagnostic and therapeutic strategy (7).

As there seems to exist a different prognosis between the several N2 subdivisions, it may be logical to make a further distinction, which has been proposed in the seventh edition of the Tumor–Node–Metastasis classification with some modifications in the eighth edition separating involvement of single from multiple nodal zones or stations (22–24). A comparison is provided in Table 2. N2 skip metastasis implies that N2 stations are involved by tumor without invasion of the intermediate N1 stations. Initial analysis for the seventh edition showed that prognosis between involvement of multiple N1 zones and single N2 zone was not different (23). In the eighth edition, the survival curves for N1b and N2a2 overlapped. N2a1 disease even had a better prognosis than N1b, although this difference was not significant (22).

**Table 2:** Subdivisions of N1-3 disease according to the seventh and eighth editions of the Tumor–Node–Metastasis classification (22, 23, 24).

Nodal subdivision	Seventh edition	Eighth edition
N1a	Single N1 zone	Single N1 station
N1b	Multiple N1 zones	Multiple N1 stations
N2a	Single N2 zone	Single N2 station
N2a1	–	Single N2 station (skip metastasis)
N2a2	–	Single N2 station (with N1 involvement)
N2b	Multiple N2 zones	Multiple N2 stations
N3	Contralateral hilar or mediastinal lymph node stations or scalene or supraclavicular lymph nodes	Contralateral hilar or mediastinal lymph node stations or scalene or supraclavicular lymph nodes

Thoracic surgeons and oncologists are encouraged to submit prospective data to the International Association for the Study of Lung Cancer (IASLC) database to obtain more reliable survival data in larger groups of patients originating from different continents (www.crab.org).

## T4N0-1M0 (Stage IIIA)

T4 disease implies a locally highly aggressive tumor with invasion of critical mediastinal organs or structures as, e.g., esophagus, carina, aorta, or left atrium. By definition, this extension is mostly beyond the limit of potential surgical resectability implying that most patients do not qualify for surgical resection. In these particular cases, it may be quite challenging to obtain a complete R0 resection according to the IASLC definition (5). When lymph nodes are involved, especially, mediastinal N2 stations, two negative prognostic factors are combined resulting in only exceptional 5-year survivors. However, several non-randomized series have shown that, in highly selected patients, long-term survival may be obtained, especially in those patients with good performance status and negative lymph nodes (25). Undoubtedly, these surgical interventions are quite complex, usually involving procedures on large vessels or carina, which require highly skilled thoracic surgeons working in a dedicated environment of a multidisciplinary team composed of medical and radiation oncologists, pulmonary physicians, radiologists, nuclear medicine physicians, pathologists, and intensive-care specialists besides thoracic surgeons. Also a specifically trained nursing and physiotherapy staff is required to detect and treat postoperative complications at an early stage (26). To obtain the best short- and long-term results, these patients should be treated in high-volume thoracic centers (25).

Most reported experience include tumors invading the superior vena cava, left atrium, carina, and intrapericardial pulmonary vessels whereby 5-year survival rates between 9 and 48% have been reported (25). Whether induction therapy may yield similar results of downstaging as in stage IIIA-N2 disease remains an open question. In some cases, induction chemotherapy or chemoradiation may be helpful for the thoracic surgeon to obtain a subsequent complete resection in order to increase overall and disease-free survival. In a Spanish phase II study, 136 patients with clinical stage IIIA or IIIB disease were treated by induction chemotherapy followed by surgical intervention (27). Complete resection was obtained in 69% of operated patients, or 48% of all assessable patients. Pneumonectomy was necessary in 41% of patients underscoring the extent of the operation that is necessary in these particular cases. Overall mortality was 7.8% and major complications occurred in 30%. In case of complete resection of a T4N0 tumor, an excellent 5-year survival rate of 53% was obtained. However, it should be noted that these were highly selected patients.

A specific category of T4 disease is those patients with ipsilateral tumor nodules in a different lobe than the primary tumor (28). They have an intermediate prognosis between patients with additional tumor nodules in the same lobe and those with distant metastases. It is usually recommended to perform a lobectomy for the largest tumor and a segmentectomy or wide wedge excision for the smallest one, although in some cases, a pneumonectomy may be required. Five-year survival rates of 22% may be obtained in this particular subset of patients (29).

At the present time, no randomized evidence on surgery for T4 disease is available; surely, such evidence will be very difficult to obtain due to the relative scarcity and heterogeneity of this patient population.

## Targeted Therapies—Immunotherapy

Newer therapeutic options include targeted therapies and immunotherapy. Targeted therapies may be given to patients with specific mutations as epidermal growth factor receptor (EGFR) mutations treated with tyrosine kinase inhibitors. Due to the good results in metastatic NSCLC, immunotherapy is currently also considered for earlier stages of lung cancer. Monoclonal antibodies, such as nivolumab, may stimulate the immune system in different ways and kill tumor cells remaining after surgery and chemotherapy. In a very recently published phase III trial, durvalumab was compared with placebo in patients with unresectable stage III NSCLC who had no evidence of disease progression after two or more cycles of platinum-based chemoradiotherapy (30). Progression-free survival was significantly longer with durvalumab than with placebo. For resectable stage III NSCLC, no randomized evidence is currently available, but there are several ongoing trials incorporating these newer therapeutic options with surgical resection. Recruiting trials incorporating targeted therapies are summarized in Table 3.

**Table 3:** Ongoing studies on stage III lung cancer incorporating targeted therapies.

NCT identifier	Status	Study title	Intervention	Phase
NCT01857271	Recruiting	Erlotinib hydrochloride before surgery in treating patients with Stage III non-small cell lung cancer (NSCLC) (EVENT)	Drug: Erlotinib hydrochloride	2
			Procedure: therapeutic conventional surgery	
			Other: laboratory biomarker analysis	
NCT02201992	Recruiting	Crizotinib in treating patients with Stage IB-IIIA NSCLC that has been removed by surgery and ALK fusion mutations (an ALCHEMIST treatment trial)	Drug: Crizotinib	3
			Other: laboratory biomarker analysis	
			Other: Placebo	
NCT02347839	Recruiting	NEoadjuvant Gefitinib followed by surgery and gefiTinib In unresectAble sTage III NSCLC with epidermal growth factor receptor mutations (NEGOTIATE)	Gefitinib-surgery-gefitinib	2

## Salvage Surgery

Salvage surgery is a relatively new concept in thoracic surgery applied to patients with recurrent or progressive disease, when no other therapeutic options are available (31). In the setting of stage III, disease salvage surgery may be indicated in patients who were initially treated by chemoradiation and in whom recurrent or progressive disease is detected at routine follow-up. These interventions should only be performed in highly selected patients who are functionally operable after thorough cardiopulmonary evaluation and be restricted to dedicated centers with a large thoracic surgical experience. In case of respiratory symptoms, fever and raised inflammatory parameters, an infected cavity may be present at the primary tumor site (32). As can be expected, these are technically complex and challenging procedures, especially when a large abscess cavity is present. At the present, time clinical series that have been published on salvage surgery for stage III disease are quite small, but they already show that an acceptable long-term survival may be obtained in patients with good performance status and low cardiopulmonary risk (33).

## Author Contributions

All authors met following criteria: substantial contributions to the conception or design of the work; or the acquisition, analysis, or interpretation of data for the work; and drafting the work or revising it critically for important intellectual content; and final approval of the version to be published; and agreement to be accountable for all aspects of the work in ensuring that questions related to the accuracy or integrity of any part of the work are appropriately investigated and resolved.

## Conflict of Interest Statement

The authors declare that the research was conducted in the absence of any commercial or financial relationships that could be construed as a potential conflict of interest.
